# Functional and dysfunctional fear of COVID-19: a classification scheme

**DOI:** 10.1186/s40163-020-00137-2

**Published:** 2021-02-05

**Authors:** Reka Solymosi, Jonathan Jackson, Krisztián Pósch, Julia A. Yesberg, Ben Bradford, Arabella Kyprianides

**Affiliations:** 1 Department of Criminology, School of Social Sciences, Williamson Building, University of Manchester, Oxford Road, Manchester, M13 9PL UK; 2 London School of Economics & Political Science, London, UK; 3 University of Sydney Law School, Sydney, Australia; 4 Jill Dando Institute of Security & Crime Science, University College London, London, UK

**Keywords:** Covid-19, Worry, Wellbeing, Re-engagement, Risk, Precautions

## Abstract

Worry about COVID-19 is a central topic of research into the social and economic consequences of the COVID-19 pandemic. In this paper, we present a new way of measuring worry about catching COVID-19 that distinguishes between worry as a negative experience that damages people’s quality of life (dysfunctional) and worry as an adaptive experience that directs people’s attention to potential problems (functional). Drawing on work into fear of crime, our classification divides people into three groups: (1) the unworried, (2) the functionally worried (where worry motivates proactive behaviours that help people to manage their sense of risk) and (3) the dysfunctionally worried (where quality of life is damaged by worry and/or precautionary behaviour). Analysing data from two waves of a longitudinal panel study of over 1000 individuals living in ten cities in England, Scotland and Wales, we find differing levels of negative anxiety, anger, loneliness, unhappiness and life satisfaction for each of the three groups, with the dysfunctionally worried experiencing the most negative outcomes and the functionally worried experiencing less negative outcomes than unworried. We find no difference between groups in compliance and willingness to re-engage in social life. Finally, we show a difference between the dysfunctionally worried compared with functional and unworried groups in perceptions of risk (differentiating between likelihood, control and consequence). This finding informs what sort of content-targeted messaging aimed at reducing dysfunctional worry might wish to promote. We conclude with some thoughts on the applicability of our measurement scheme for future research.

## Introduction

The 2019 novel coronavirus (COVID-19) has affected many aspects of life globally. One consequence has been the surge of public anxieties and worries in some parts of the world (Lin 2020), with significant levels of fear of catching COVID-19 being recorded in the UK (Fancourt et al. 2020), Germany (Gerhold 2020), Iran (Ahorsu et al. 2020), India (Roy et al. 2020) and China (Lin 2020). Worry about catching COVID-19 has been associated with negative outcomes such as poor mental health (Sloan et al. 2020) and higher levels of prejudice (Lin 2020; Roy et al. 2020). It may also be related to reluctance to re-engage with economic and social activities as lockdown eases (Shaw et al. 2020).

In and of itself, worry (thinking and feeling anxious about actual or potential problems) can damage people’s mental health and quality of life. Yet, worry can also stimulate care and precaution. The type of worry that helps people to develop coping strategies has been described in the fear of crime literature as “functional fear”; people use adaptive emotions and precautionary activities to help guard themselves against the cause of their worry (Jackson and Gray 2010). “Dysfunctional fear”, by contrast, involves people worrying about crime and reporting that their quality of life is negatively affected by this worry and/or their precautionary behaviour. The word “fear” used here as a marker for worry and anxiety about victimization risk (Farrall et al. 2009).

In this paper we explore whether these groupings exist for worry about COVID-19. Drawing on self-reports, we classify research participants based on whether they worry, whether that worry affects their quality of life, whether they take precautions, and whether those precautions make them feel safer or have an impact on their quality of life. We then consider whether membership of these groups is associated with different outcomes, both negative (e.g. negative affect) and positive (e.g. compliance and willingness to re-engage with social and economic life). Finally, we consider a possible intervention to address those experiencing dysfunctional fear, exploring who messaging should target, but also what content that messaging might consider. To achieve this, we look at differences in risk perception between the three groups, and speculate on the type of information that might target people’s perceived likelihood, perceived consequences, and sense of control. We argue that the dysfunctional group would be best served by targeted messaging and time and effort from governments and other organisations in order to reduce their worry, or, to shift them to the “functionally worried” group (Lee et al. 2020).

### Worry about COVID-19

COVID-19 is an infectious disease caused by a newly discovered coronavirus. To date (5th January 2021) there have been 84 million confirmed cases and 1,160,650 confirmed deaths across 226 countries and territories (World Health Organisation 2020a). Understandably, such an unprecedented global health crisis has evoked worry and anxiety in large segments of the population of countries across the world.

Worry about COVID-19 is a topic of interest in research and policy as it is thought to (a) stimulate adherence with public health measures (good hygiene, social distancing etc.), (b) have negative consequences on mental and physical health, and (c) prevent people from re-engaging with social and economic life once government restrictions of such activities are eased. This concern has spurred various initiatives. For example, the World Health Organisation has supported a range of projects aimed to reduce COVID-19 related stress, including, a telephone counselling service in Turkey (World Health Organisation 2020b).

Research into worry about COVID-19 has focused on establishing a way to gauge prevalence of worry (Ahorsu et al. 2020) and explore some of the possible consequences of worry, e.g. impact on wellbeing (Fancourt et al. 2020; Sloan et al. 2020), behaviour changes (Gerhold 2020), and prejudice against groups perceived as associated with the spread of the virus (Lin 2020; Roy et al. 2020). It is our contention, however, that the literature would benefit from engaging with previous work in victimization worry and risk perception, particularly the understanding that while worry can be a negative and debilitating experience that discourages healthy re-engagement with the world, it can also be a problem-solving activity, directing people’s attention to problems and encouraging them to act accordingly.

The distinction between ‘adaptive’ worry and ‘corrosive’ worry is illustrated in a current debate in the literature on the nature and impact of fear of COVID-19 (Ahorsu et al. 2020; Harper et al. 2020). Ahorsu et al. (2020) assessed the scaling properties of items such as “I cannot sleep because I am worried about getting coronavirus-19”, “It makes me uncomfortable to think about coronavirus-19” and “When watching news and stories about coronavirus-19 on social media, I become nervous or anxious”. Because the resulting scale was moderately and positively correlated with depression, anxiety, perceived infectability and germ aversion, what seems to emerge is a continuum that, at one extreme, is something akin to psychopathology, i.e. something that is part of a spectrum of emotional conditions characterized by negative affect, including phenomena such as depression, anxiety, and phobias.

Importantly for the current study, however, Harper et al. (2020) fielded the same scale on a new sample, and found that people who scored higher on the scale were more likely to practice positive public health behaviours like social distancing. They argued that rather than measuring some kind of pathological “fear”, the scale may instead be tapping into adaptive negative emotions that help guide people to respond to dangerous situations. This is relevant. Psychologists have long recognised the problem-solving function of worry in the anticipation of future problems and risks (Gladstone and Parker Gladstone and Parker 2003a, b). Worry can be a routine and mostly acceptable activity that occurs more or less daily, about various issues. It transpires mostly in the form of thoughts with a narrative course, stimulates problem-solving activity, and is an indicator of high levels of responsibility (Tallis et al. 1994; Berenbaum 2010).

Such thinking has already been applied to worry about crime (Jackson and Gray 2010; Gray et al. 2011). Fear of crime can have adverse emotional effects upon people, inducing a feeling of isolation and vulnerability and general loss in personal well-being (Hale 1996), motivating people to remain indoors more than they would wish or to avoid certain places (Moore and Trojanowicz 1988); in extreme cases, it can be destructive and paralysing. But people and communities have the potential to convert worry about crime into constructive action; people who worry in such a pro-active, non-detrimental manner can be described as experiencing “functional fear” (Lee et al. 2020).

### A typology of worry about COVID-19

How, then, should we conceptualise thinking and feeling anxious about actual or potential problems related to contracting COVID-19? Macleod et al. 1991 define worry as a cognitive phenomenon ‘…concerned with future events where there is uncertainty about the outcome, the future being thought about is a negative one, and this accompanied by feelings of anxiety.’ We proceed on the basis that worry can be differentiated into functional and dysfunctional, based on its nature, effects and how it is managed by people (Tallis et al. 1994; Gladstone and Parker 2003b). On the one hand, worry can be constructive, helping people to solve potential problems in numerous life domains, e.g. educational performance, finances, and job prospects. On the other hand, worry can be destructive, with people becoming preoccupied with negative information and future unpleasant outcomes, hyper-vigilant in scanning for salient material relating to threat, engaged in the over-estimation of risk, and so forth (Butler and Mathews 1983; Butler and Mathews 1987; Tallis and Eysenck 1994).

We build on work into functional fear of crime. Jackson and Gray 2010 separated people into these groups of functional and dysfunctional worry about falling victim of crime. The measurement strategy they used classified people into the ‘functional fear of crime’ group when they said that they were worried about crime, that they took precautions against crime, that these precautions made them feel safer, and crucially, that they thought that their quality of life was reduced neither by their worry nor by their precautions. The implication here is that functional worry motivates a behavioural response that helps people manage any insecurity in a way that their quality of life is unharmed. By contrast, ‘dysfunctional fear’ was assumed to be present when people said that their quality of life was reduced either by worry or precautionary behaviour (or both). Jackson and Gray 2010 found that victimization experience was associated with dysfunctional worry, but not with functional worry. In another study, Gray and colleagues found that those experiencing ‘dysfunctional worry’ were at greater risk than those in the other worry categories of experiencing health problems and seeing their neighbourhood as disorderly and lacking in collective efficacy (Gray et al. 2011).

Our approach to measurement follows the same template. We build on psychological work on worry using a similar empirical approach as Jackson and Gray 2010, which means bringing into focus people’s precautionary behaviour and sense of impact on their own quality of life. We ask research participants whether they have or have not worried about catching COVID-19 (we draw on data from waves 2 and 3 of a panel study, with 64% in wave 2 saying yes), whether they take precautions against COVID-19 (91% in wave 2 said yes), and whether the precautions they took made them feel safer (81% in wave 2 said ‘moderately’, ‘quite a bit’ or ‘very much’). Then, based on whether research participants said that their quality of life was reduced by their worries and/or precautions, we classify research participants into one of three groups: (a) unworried, (b) the functionally worried, and (c) the dysfunctionally worried.

We then explore whether worry about COVID-19 is associated with experience with COVID-19 like infection or infection of a loved one, and other related issues such as losing a job or experiencing economic hardship due to government restrictions aimed to curb the spread of COVID-19. We also assess whether there is a difference in negative emotions between the different types of worry about COVID-19, and whether worry about COVID-19 is related to compliance with government restrictions aimed to curb the virus, as well as subsequent re-engagement in social and economic activities. Graham et al. (2020) found that personal fear of COVID-19 significantly predicted compliance with social distancing ordinances. Those who were more afraid of COVID-19 were more likely to say they would engage in social distancing. They also found that COVID-related cognitions (perceived risk) and emotions (fear) had different effects on compliance. However, Wright et al. 2020 find evidence that increased confidence in government to tackle the pandemic is longitudinally related to higher compliance, but little evidence that factors such as mental health and wellbeing, worries about future adversities, and social isolation and loneliness are related to changes in compliance. In light of this mix of findings, it is important to further explore this association.

### Risk perception and change in worry group

Finally, to help inform effective messaging designed to move people from one group to another (specifically from the dysfunctional group to the functional group), we consider what sort of perceptions might mean someone is in one group over the other. Much fear of crime literature focuses on people’s demographic characteristics (age, gender, race), and this is also the case in fear of COVID-19 literature. But fear of crime is transitory and situational, meaning that people move between states of worry. Studies following people over time have found that their worry levels change as they experience various different situations which might make them worry (Chataway et al. 2017; Solymosi et al. 2015). It is possible that people also move between ‘functional’, ‘dysfunctional’ worry, and unworried groups over time. Due to the longitudinal survey design, we can examine this for worry about COVID-19, to see how people might shift between experiencing different types of worry over time.

If people do indeed shift, then that lends support to initiatives aimed to help people out of dysfunctional states of worry into functional fear. But the question remains what sort of messaging might be helpful. Fear of crime scholarship has benefited from engagement with research in the psychology of risk by establishing that perceptions of the likelihood, consequence and controllability of criminal victimization predict levels of fear of crime (Jackson 2009). To establish whether this is also the case for fear of COVID-19, we apply these measures of risk perception to concerns about contracting the virus: namely, people’s perceived likelihood of the event happening to them, the perceived control that they have over the occurrence of the event, and the perceived severity of the consequences of the event happening. In relation to worry about crime, it seems that the more an individual judges the likely consequences to be severe, and the less control they feel they have over this risk, the higher the expected value of perceived likelihood (Jackson 2011). If similar risk perceptions are also associated with worry about COVID-19, it might be an idea to target these perceptions with information campaigns based on notions of likelihood and/or control and/or consequence, to reduce dysfunctional worry.

## Data and methods

### Sample

We use data collected as part of the ‘Policing the pandemic’ longitudinal study. The surveys were hosted on Qualtrics and fielded on the online platform Prolific Academic. Prolific is similar to other crowdsourcing platforms such as Mechanical Turk but has a larger, more diverse pool of UK participants. In line with Prolific recruitment protocols, participants received compensation for their time. We use data from the second and third waves collected between 11 and 14 May (wave 2) and 1–5 June (wave 3) 2020 (The first wave did not ask questions about worry). The second wave was fielded the day after UK prime minister Boris Johnson’s address to the nation that announced an easing of the initial lockdown restrictions. These changes involved revised messaging from ‘stay at home’ to ‘stay alert’ as well as some rule changes (e.g. allowing more outdoor activities within and contact between households), and came into force on 13 May. The data collection of the third wave took place 10 days after the nation learnt about the lockdown breach by Dominic Cummings, the prime minister’s chief advisor, which was followed by a national outcry. The data collection for wave 3 also coincided with further easing of the restrictions, including permitting up to six people to meet outside, the reopening of some childcare facilities, and so on. According to the data by the Office of National Statistics, on the week of survey wave 2, 3810 people died with COVID in the UK; this dropped to 1588 COVID deaths by the week of wave 3. A total of 1100 people participated in wave 2 of the study, and 1019 in wave 3. Respondents lived in ten metropolitan areas across the UK (Birmingham, Cardiff, Edinburgh, Glasgow, Leeds, Liverpool, Manchester, Newcastle, Sheffield, and London). Quota weights were calculated^1^ for gender and age. Although quota sampling is not probabilistic in nature, the stratification involved with the sampling strategy meant that after the data collection quota weights could adjust the results making them largely representative of the ten cities in our sample.

### Variables

This section details how we operationalise the key concepts discussed above. The specific questionnaire items are in Appendix 1.

### Worry and precautionary activity

In order to classify worry about COVID-19, we use answers to questions adapted from fear of crime questions (e.g. see Jackson et al. 2009), asking people whether they had felt worried about getting COVID-19 in the past 3 weeks, and to what extent their quality of life was reduced by this worry. We note that we use the phrases “functional fear” and “dysfunctional fear” because they are good phrases, but we do not claim to be measuring fear—we ask research participants about their “worry”, leaving them to interpret what “worry” means. By explicitly stating ‘worry’ and ‘worried’ in the question wording, we make sure to measure worry as both a cognitive and emotional phenomenon.

To measure whether people take precautions and how these affect them, we asked questions based on precautions people take against crime victimisation (e.g. see Newton et al. 2020), asking whether they take precautions, and to what extent these affect their quality of life and make them feel safer. Regarding the definition of ‘quality of life’ we left it up to participants to define what this means to them, as the question asked directly to what extent either worry or precautions ‘reduce your quality of life’.

### Previous experience

To match ‘previous victimisation’ in fear of crime studies, we asked about previous experience with COVID-19. This included whether participants had had COVID-19 themselves or whether they had been affected by any of the following: themselves or a member of their household losing their job or being unable to work, being unable to pay bills, access sufficient food or required medication, having been evicted or lost accommodation otherwise, and having someone they are close to go in hospital or die as a result of COVID-19. Based on these measures, a summative score of how much the person had been affected by COVID-19 was calculated, where higher scores indicate they were more severely affected. This is not a construct as such, rather a sum of negative life experiences (possibly unrelated to one another) caused by COVID-19.

### Emotions

To assess the extent to which mental and emotional wellbeing is associated with worry we asked people about how strongly they had felt anxiety, anger, loneliness, and happiness in the previous day. We also asked people overall how satisfied they are with their life and to what extent they feel that the things they do in life are worthwhile. Those who answered they felt anxiety/anger/loneliness “Not at all” were coded as “No”, everyone else as “Yes” to experiencing these outcomes. For happiness, those who answered “Not at all” were coded as “Yes” to never having felt happy, and for Worthwhile and Satisfied we coded “Not at all…” feeling that the things they do in their lives were worthwhile or “Not at all…” satisfied with their lives as “Yes” to feeling not satisfied and not worthwhile. As a result, a positive (“Yes”) outcome for each respective emotions indicator represents a bad emotional experience. We also created a summative emotion score, where answers were re-coded to run from 1 to 5, with higher scores representing a worse emotional outcome. A Cronbach’s alpha of 0.88 indicates good internal consistency among these indicators.

### Compliance and reengagement

We also asked people the extent to which they engaged in the following behaviours: socialised in person with friends or relatives they do not live with; went for a walk or run or cycle and spent time sitting somewhere to relax; and travelled for leisure. In wave 2 these behaviours were in breach of the lockdown restrictions put in place by government, so participating in these events reflected a lack of compliance. However, in wave 3, these behaviours were once again allowed. As a result, people in the UK were being encouraged to re-engage in some semblance of normal life–they were allowed to stop and rest during or after walking, running and cycling, they were allowed to travel for leisure, and they were allowed to socialise in groups of 6 in England or 8 in Scotland. Thus, changes in self-reported behaviour betweens waves 2 and 3 represented the extent to which research participants were re-engaging with the social world. We calculated a re-engagement score by taking the score for the compliance item in wave 2 and subtracting it from the sum of the compliance items in wave 3. This means higher scores indicate more re-engagement: A score of 0 represents no change in engagement, a negative score represents more engagement in wave 2 than wave 3 (less engagement after easing of lockdown), and a positive score signals more participation in the activity in wave 3 than wave 2 (re-engagement). Similar to impact of COVID-19 score, this is not a construct as such, rather a mock-count using a formative approach to measurement adding up people’s answers to create an index.

### Control variables

Of course, other factors besides worry might affect compliance and reengagement, so we included measures of the following in our modelling: knowledge of the virus (self-rated knowledge level of the virus), their perceived risk of sanctions for breaching lockdown restrictions (how likely they think it is to be caught and sanctioned for each activity), expressive function of the law (whether they think it was right or wrong to make social distancing a legal requirement, and whether doing so clarifies the restrictions and sends the message of its importance in fighting the pandemic), social norms (whether people think it is important to follow social distancing guidelines and if they perceive their community to also think this is important), feelings of duty to obey the police and follow the law, and their normative alignment with the police and the law.

We also asked for demographics, such as age, gender, ethnicity, and whether they held employment that considered them to be ‘key workers’.

### Risk perception

Finally, to measure perception of risk, we used three risk related questionnaire items adapted from perceived victimization risk questions (Jackson 2009) to measure perceived likelihood (how likely they think it is to catch COVID-19 in next 3 weeks), perceived severity of consequences (how severe they expect the consequences to their health to be), and perceived control (to what extent they believe they can control whether or not they catch COVID-19 in the next 3 weeks).

## Results

### Defining and measuring worry about COVID-19

In wave 2 just over one-third (34%) of people said that “No”, they had not worried about getting COVID-19 in the past 3 weeks. From those who said “Yes”, there was variation in self-reported frequency and intensity of worry. 35% of people who were worried experienced this “Once or twice” in the last 3 weeks, while 21% worried more than 10 times in this timeframe. On the last occasion, 58% said they “felt fairly worried” or “very worried”.

First, individuals were classified as unworried if they reported being unworried about catching COVID-19: it did not matter if they took precautions that made them feel safer, or if their quality of life was reduced by their precautions; if they reported being unworried they were simply classified as unworried.

To be classified in the functional worry group, respondents must have met three conditions: (a) they must have reported being worried about catching COVID-19; (b) they must have taken precautions that made them feel safer; and (c) they must have judged their quality of life to be unaffected by either their worries or their precautions. Importantly, we assume that the worry process partly motivates these beneficial precautions; as Tallis and Eysenck 1994 argue, worry can play a problem-solving role in people’s lives by stimulating action and helping them deal with uncertain future events. Finally, to be classified in the dysfunctional worry group, respondents must have reported being worried about COVID-19 but also that their quality of life was reduced by either their worries or their precautions (or both), or their precautions to not have made them feel safer.

To generate the three groups, Tables 1 and 2 break down the sample. Overall 35% (n = 401, weighted n = 312) of respondents were unworried. The other two categories—functional worry and dysfunctional worry—are subsets of the remaining 65% of respondents (n = 690, weighted n = 581). Of these, 4% (n = 34, weighted n = 25) took no precautions, 93% (n = 641, weighted n = 541) took precautions and felt safer as a result, and 1% (n = 5, weighted n = 6) took precautions but did not feel safer as a result (Table 1). We make this distinction because of the central role that beneficial precautionary activity plays in the functional/dysfunctional distinction.

**Table 1:** Worry about COVID-19, precautionary activity, and impact of feelings of safety

	Takes no precautions	Takes precautions and feels safer as a result	Takes precautions and does not feel safer as result	Did not answer whether precaution made them safer	Total (row percentages)
Unworried, *n *= 401 (35%)	–	–	–	–	–
Worried, *n *= 690 (64%)	34 (4%)	641 (93%)	5 (1%)	10 (2%)	100%

Source: Unweighted data from wave 2 of the Policing the pandemic

**Table 2:** Precautionary activity and impact on quality of life impact amongst those who are worried about catching COVID-19

	Combined effect of worry about COVID-19 and precautions against COVID-19 on quality of life		
	None or little effect of both	Some or strong effect of either or both	Total (column %)
Took precautions and felt safer as a result	203^a^	438^b^	641 (93%)
Did not take precautions or took precautions and did not feel safer as a result	3^b^	36^b^	39 (6%)
Took precautions but did not answer if felt safer	–	10^b^	10 (1%)
Total (row %)	206 (30%)	484 (70%)	690 (100%)

Source: Unweighted data from wave 2 of the Policing the Pandemic research project

^a^This cell makes up the “functional fear” group

^b^These cells make up the “dysfunctional fear” group

Table 2 takes the categorisation process one step further by also considering whether the worry or the precautions had an effect on people’s quality of life. Overall 37% (n = 206, weighted n = 203) said their quality of life was not affected by either precautions or worry, 33% (n = 236, weighted n = 181) said they were affected by both, 12% (n = 82, weighted n = 64) said their quality of life was reduced by their worry but not the precautions, and 18% (n = 122, weighted n = 99) said this was the other way around (precautions but not the worry).

By cross-tabulating precautionary activity with levels of impact on quality of life, we can identify the functionally worried and the dysfunctionally worried. The cell to highlight is top-left (Table 2). This represents the functionally worried—the subset of the sample who were worried about COVID-19, who took precautions that made them feel safer, and whose quality of life was not reduced by either worry or precaution. The other three cells comprise the dysfunctionally worried group.

Bringing this classification process to a close, we found that just over one-third (35% (n = 401, weighted n = 312)) of the sample were unworried, about one-in-five (22% (n = 203, weighted n = 198)) were functionally worried, and just over two-in-five (43% (n = 487, weighted n = 383)) were dysfunctionally worried (Table 3).

**Table 3:** Worry about COVID-19, precautionary activity, and the quality of life

	*n*	Total %
Unworried	401	37
Functionally worried	203	19
Dysfunctionally worried	487	45

Source: Unweighted data from wave 2 of the Policing the pandemic

### Previous experience

We consider whether the person had COVID-19 themselves or not and draw on our measure of how much COVID-19 had affected their lives by tallying the many negative outcomes we asked about. In total, 14% (183) had themselves lost their job or were unable to work, 10% (118) had a member of their household lose their job or be unable to work, 6% (67) reported being unable to pay bills, 0.4% (5) couldn’t access sufficient food, 5% (59) couldn’t access required medication, 2% (28) had been evicted or otherise lost their accommodation, 3% (36) saw someone they are close to go in hospital and 3% (35) saw someone close die as a result of COVID-19. Some 171 people reported suspecting themselves having COVID-19.^2^

We see that those who have had (or thought themselves to have had) contracted COVID-19 already are more likely to be in the functionally worried group (vs unworried). On the other hand, those scoring higher in the COVID-19 effect measures are those in the ‘dysfunctional worry’ group (Table 4).

**Table 4:** Previous experience with COVID-19, and worry group

	Dependent variable			
	Functional Worry	Dysfunctional Worry	Functional Worry	Dysfunctional Worry
	(1)	(2)	(3)	(4)
*Had COVID-19*	*2.439*	*1.012*	*2.076*	*0.936*
	* (0.293)*	* (0.178)*	* (0.348)*	* (0.215)*
	p = 0.003^***^	*p = 0.946*	p = 0.036^**^	*p = 0.757*
*Affected by COVID-19*	*0.979*	*1.465*	*0.860*	*1.458*
	* (0.127)*	* (0.091)*	* (0.162)*	* (0.108)*
	*p = 0.866*	p = 0.00003^***^	*p = 0.349*	p = 0.0005^***^
Age 25–44^a^			0.889	1.238
			(0.267)	(0.207)
			p = 0.660	p = 0.303
Age 45–64^a^			2.264	1.611
			(0.351)	(0.304)
			p = 0.020^**^	p = 0.117
Age 65+^a^			2.081	2.085
			(0.760)	(0.657)
			p = 0.335	p = 0.264
Female (ref: Male)			1.252	1.310
			(0.217)	(0.170)
			p = 0.301	p = 0.113
White (ref: BAME)			0.883	1.113
			(0.298)	(0.234)
			p = 0.676	p = 0.648
Is keyworker			0.978	0.964
			(0.040)	(0.031)
			p = 0.577	p = 0.233
Cardiff^b^			1.889	1.256
			(0.667)	(0.450)
			p = 0.341	p = 0.613
Edinburgh^b^			2.968	1.698
			(0.626)	(0.425)
			p = 0.083^*^	p = 0.213
Glasgow^b^			2.392	0.841
			(0.610)	(0.424)
			p = 0.153	p = 0.683
Leeds^b^			2.445	2.138
			(0.630)	(0.410)
			p = 0.156	p = 0.064^*^
Liverpool^b^			2.759	1.532
			(0.622)	(0.419)
			p = 0.103	p = 0.309
London^b^			2.630	1.282
			(0.533)	(0.336)
			p = 0.070^*^	p = 0.460
Manchester^b^			2.730	1.025
			(0.600)	(0.414)
			p = 0.094^*^	p = 0.953
Newcastle^b^			1.654	0.950
			(0.722)	(0.480)
			p = 0.486	p = 0.915
Sheffield^b^			3.390	0.975
			(0.607)	(0.435)
			p = 0.045^**^	p = 0.954
Constant	0.016	0.969	0.014	0.922
	(1.152)	(0.686)	(1.510)	(0.933)
	p = 0.0004^***^	p = 0.963	p = 0.005^***^	p = 0.932
Akaike Inf. Crit.	2182.704	2182.704	1626.462	1626.462

Multinomial logistic regression model estimated using R (package nnet). Source: Wave 2 of Policing the pandemic. Total n = 1058

* p < 0.05, ** p < 0.01, *** p < 0.001

^a^Reference category: Age 16–24

^b^Reference category: Birmingham

### Negative outcomes on emotional well being

**Fig. 1:**
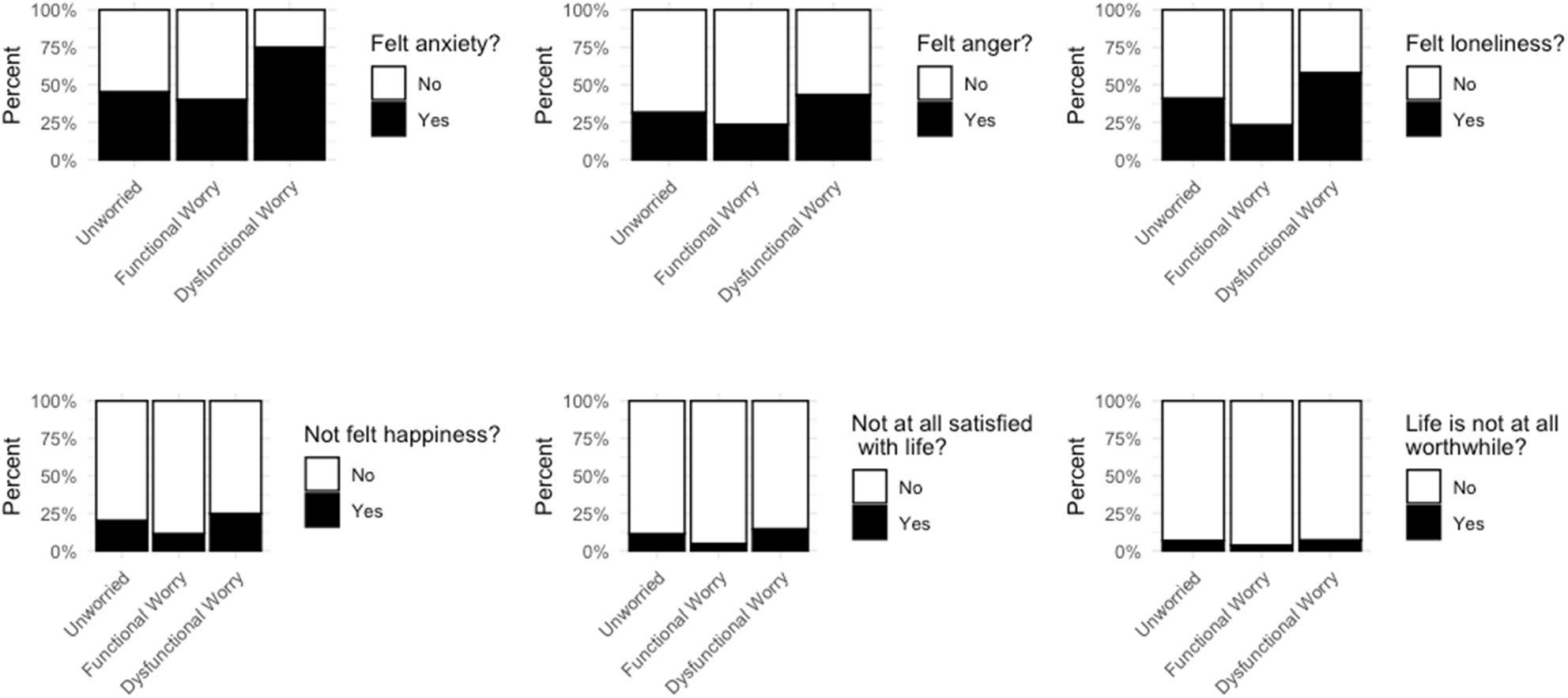
Emotional wellbeing outcomes by worry group

We find that separating out the types of worry, and comparing to the unworried, shows that for all our measures of emotional wellbeing those in the ‘dysfunctionally worried’ group report more negative emotions than those who are not worried, while those in the ‘functional’ worry group do not. In fact, on all outcomes, those in the functionally worried group report fewer negative emotional outcomes than the unworried, while dysfunctional worry group report more (Fig. 1).

Separating out the functional and dysfunctional worry here highlights that it is those who are experiencing ‘dysfunctional worry’ that are more affected by these negative outcomes, and not the ‘functional worry’ group.

Modelling the outcome variable to the summative score on negative emotional outcomes shows that those in the functional worry group score lower on this negative emotion score than unworried people, while those in the dysfunctional group score higher, i.e. have worse wellbeing outcomes (Table 5).

**Table 5:** Negative emotional wellbeing outcomes by worry group

	Dependent variable	
	Emotion score	
	(1)	(2)
Functional worry^a^	*0.913*	*0.936*
	(0.026)	(0.031)
	p = 0.0005^***^	p = 0.034^**^
Dysfunctional worry^a^	*1.164*	*1.162*
	(0.021)	(0.025)
	p = 0.000^***^	p = 0.000^***^
Had COVID-19		0.977
		(0.031)
		p = 0.443
Affected by COVID-19		1.061
		(0.016)
		p = 0.0002^***^
Age 25–44^b^		0.946
		(0.029)
		p = 0.057^*^
Age 45–64^b^		0.885
		(0.032)
		p = 0.0002^***^
Age 65+^b^		0.826
		(0.044)
		p = 0.00002^***^
Female (ref: Male)		1.007
		(0.022)
		p = 0.761
White (ref: BAME)		1.097
		(0.039)
		p = 0.017^**^
Is keyworker		1.010
		(0.004)
		p = 0.027^**^
Cardiff^c^		0.955
		(0.049)
		p = 0.355
Edinburgh^c^		0.900
		(0.051)
		p = 0.039^**^
Glasgow^c^		0.985
		(0.049)
		p = 0.752
Leeds^c^		1.003
		(0.049)
		p = 0.958
Liverpool^c^		0.957 (0.051)
		p = 0.393
London^c^		0.997
		(0.050)
		p = 0.950
Manchester^c^		1.018
		(0.050)
		p = 0.728
Newcastle^c^		1.005
		(0.050)
		p = 0.917
Sheffield^c^		0.910
		(0.052)
		p = 0.069^*^
Constant	14.312	14.093
	(0.016)	(0.134)
	p = 0.000^***^	p = 0.000^***^
Observations	1058	780
Log Likelihood	− 2443.521	− 1619.934
theta	151.136^*^ (77.824)	114,651.800 (1,204,052.000)
Akaike Inf. Crit.	4893.042	3279.869

Negative binomial generalized linear model estimated using R (package MASS). Source: Wave 2 of Policing the pandemic. Total *n* = 1058

* p < 0.05, ** p < 0.01, *** p < 0.001

^a^Reference category: Unworried

^b^Reference category: Age 16–24

^c^Reference category: Birmingham

### Lockdown compliance/re-engaging with social and economic activities

We next consider whether worry is a factor in shaping people’s compliance with lockdown regulations, and whether worry discourages people from re-engaging with social and economic activities once these restrictions are eased. We asked people a series of questions related to compliance as well as questions about other factors which may influence such behaviours (see “Variables” section in “Data and methods”). When we consider the effect of being in either the functionally or dysfunctionally worried group, while controlling for these other possible predictors of compliance with social distancing (Table 6) and re-engagement with the social world (Table 7), we find that functional worry does play a role in social distancing: people who are functionally worried are more likely to report frequent compliance with social distancing than people who are unworried or dysfunctionally worried. Neither type of worry seems to play a significant role in overall compliance.

**Table 6:** Poisson model of self-reported non-compliance with social distancing

	Coeff.	Std error	*P* value	95% CI	
Constant	0.26	0.31	0.39	− 0.35	0.88
Knowledge about COVID-19	− 0.11	0.07	0.10	− 0.23	0.02
Deterrence (perceived chance of police intervention in the event of non-compliance)^a^	− 0.02	0.05	0.76	− 0.11	0.08
Perceived legal legitimacy^a^	− 0.17	0.07	0.01	− 0.31	− 0.03
Perceived police legitimacy^a^	0.16	0.07	0.01	0.04	0.31
Social norms regarding social distancing^a^	− 0.47	0.07	<0.001	− 0.61	− 0.33
Expressive function of the law^a^	− 0.22	0.05	<0.001	− 0.31	− 0.13
Functional worry about catching COVID-19	− 0.40	0.12	<0.001	− 0.64	− 0.16
Dysfunctional worry about catching COVID-19	− 0.13	0.10	0.18	− 0.32	0.06
Age gender interaction: 16–24 female^b^	− 0.42	0.17	0.02	− 0.76	− 0.08
25–44 male^b^	− 0.23	0.14	0.11	− 0.52	0.054
25–44 female^b^	− 0.48	0.14	<0.001	− 0.75	− 0.21
45–64 male^b^	− 0.38	0.19	0.05	− 0.76	− 0.01
45–64 female^b^	− 0.98	0.21	<0.001	− 1.40	− 0.56
65 + male^b^	− 0.91	0.70	0.19	− 2.29	0.47
65 + female^b^	− 0.99	0.67	0.14	− 2.30	0.32
City: Cardiff^c^	− 0.11	0.22	0.64	− 0.55	0.33
Edinburgh^c^	− 0.28	0.21	0.18	− 0.70	0.13
Glasgow^c^	− 0.11	0.21	0.59	− 0.51	0.29
Leeds^c^	− 0.09	0.20	0.67	− 0.31	0.48
Liverpool^c^	− 0.05	0.21	0.82	− 0.46	0.36
London^c^	0.23	0.16	0.14	− 0.08	0.53
Manchester^c^	− 0.03	0.20	0.87	− 0.42	0.36
Newcastle^c^	− 0.30	0.23	0.19	− 0.75	0.14
Sheffield^c^	− 0.24	0.23	0.30	− 0.69	0.21
None of these^c^	0.04	0.22	0.84	− 0.38	0.47

Poisson regression model estimated using Stata 15. Source: Wave 3 of Policing the pandemic. Total *n* = 1015

*OR* odds ratio, *CI* confidence intervals

* p < 0.05, ** p < 0.01, *** p < 0.001

^a^Component scores saved from principal components analysis

^b^ Reference category: 16–24 male

^c^Reference category: Birmingham

**Table 7:** Linear model of change in self-reported ‘lockdown compliance’ between waves 2 and 3 (high scores = less ‘compliance’)

	Coeff.	Std error	P-value	95% CI	
Constant	1.28	0.61	0.03	0.09	2.47
Knowledge about COVID-19 at wave 2	− 0.01	0.15	0.93	− 0.30	0.27
Deterrence (perceived chance of police intervention in the event of non-compliance) at wave 2^a^	− 0.12	0.11	0.27	− 0.33	0.09
Perceived legal legitimacy at wave 2^a^	0.14	0.14	0.31	− 0.13	0.42
Perceived police legitimacy at wave 2^a^	− 0.03	0.15	0.85	− 0.32	0.26
Social norms regarding social distancing at wave 2^a^	− 0.12	0.11	0.29	− 0.34	0.10
Expressive function of the law at wave 2^a^	0.27	0.11	0.01	0.06	0.49
Functional worry about catching COVID-19 at wave 2	− 0.07	0.21	0.74	− 0.35	0.49
Dysfunctional worry about catching COVID-19 at wave 2	− 0.02	0.17	0.92	− 0.31	0.34
Knowledge about COVID-19 at wave 3	− 0.01	0.15	0.96	− 0.30	0.28
Deterrence (perceived chance of police intervention in the event of non-compliance) at wave 3^a^	− 0.17	0.10	0.08	− 0.36	0.02
Perceived legal legitimacy at wave 3^a^	− 0.19	0.15	0.20	− 0.48	0.10
Perceived police legitimacy at wave 3^a^	0.25	0.16	0.12	− 0.07	0.56
Social norms regarding social distancing at wave 3^a^	− 0.02	0.11	0.84	− 0.24	0.19
Expressive function of the law at wave 3^a^	− 0.29	0.11	0.01	− 0.50	− 0.07
Age gender interaction: 16–24 female^b^	0.40	0.35	0.25	− 0.28	1.08
25–44 male^b^	− 0.10	0.32	0.75	− 0.73	0.53
25–44 female^b^	0.27	0.30	0.37	− 0.31	0.85
45–64 male^b^	− 0.13	0.38	0.74	− 0.89	0.63
45–64 female^b^	− 0.50	0.35	0.16	− 1.19	0.19
65 + male^b^	− 0.59	0.91	0.52	− 2.37	1.20
65 + female^b^	− 0.09	0.70	0.90	− 1.47	1.29
City: Cardiff^c^	− 0.64	0.37	0.08	− 1.36	0.08
Edinburgh^c^	− 0.30	0.35	0.40	− 0.99	0.40
Glasgow^c^	0.01	0.36	0.98	− 0.69	0.72
Leeds^c^	0.62	0.36	0.08	− 0.08	1.33
Liverpool^c^	− 0.09	0.37	0.80	− 0.82	0.63
London^c^	0.14	0.29	0.65	− 0.44	0.71
Manchester^c^	0.46	0.36	0.20	− 0.24	1.16
Newcastle^c^	0.35	0.38	0.36	− 0.40	1.10
Sheffield^c^	0.46	0.37	0.22	− 0.27	1.20
None of these^c^	− 0.05	0.39	0.91	− 0.81	0.72

Linear regression model estimated using Stata 15. Source: Waves 2 and 3 of Policing the pandemic. Total *n* = 1980

*OR* odds ratio, *CI* confidence intervals

* p < 0.05, ** p < 0.01, *** p < 0.001

^a^Component scores saved from principal components analysis

^b^Reference category: 16–24 male

^c^Reference category: Birmingham

### Risk perception

We now turn to whether it is possible for people to move between worry groups, and what might motivate them to do so. Previously we found that some factors such as previous experience with COVID-19 and the extent to which it has negatively affected people’s lives are related to which group they belong to (see ‘Previous experience’ section). Yet, this is not something that lends itself readily to practical interventions aimed to nudge people from one group to the other.

**Fig. 2:**
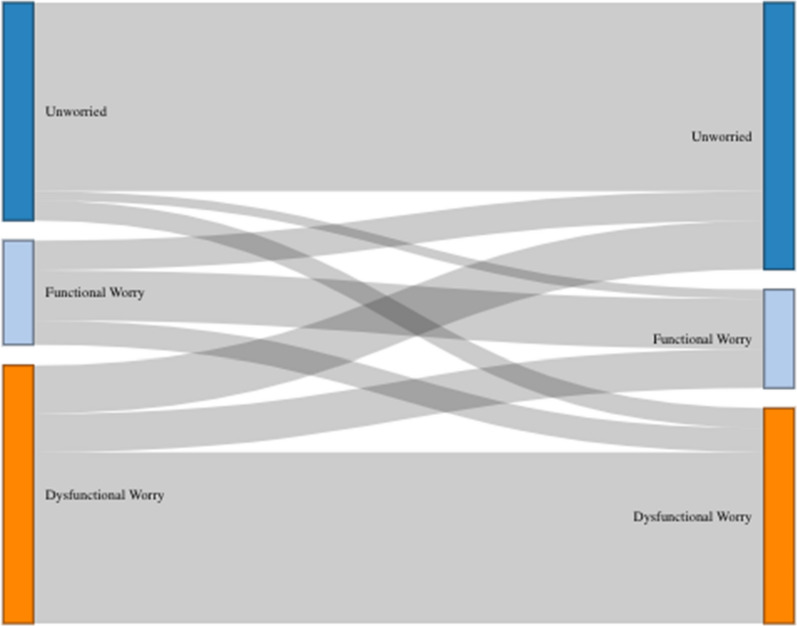
Movement between Functional, Dysfunctional, and Unworried worry groups between waves 2 and 3 of the panel survey

Instead, here we explore how perceived risk might differ between the worry groups as this might be something that targeted messaging might better be able to influence. First, we need to see whether people move between worry groups, or whether this is a static attribute. The longitudinal nature of this survey allows us to assess this. While most people stayed in the same group between the two waves (71%), almost a third (29%) moved between categories (Fig. 2).

Clearly it is possible for people to move out of this dysfunctional worry category, so messaging targeted at this group to encourage this shift might be a helpful policy intervention to explore. But what sort of content might be useful? Would they be messages based on control: what people can do to manage their risk? Would they be messages based on consequence: would they stress the low relative risk of serious health outcomes among, say, younger people?

As mentioned earlier, in relation to fear of crime, risk perception in terms of the perceived likelihood of victimization, control over this likelihood, and the perceived severity of the consequences all play a role in people’s worry (Jackson 2011). It is possible that these also affect belonging to worry about COVID-19 groups.

**Fig. 3:**
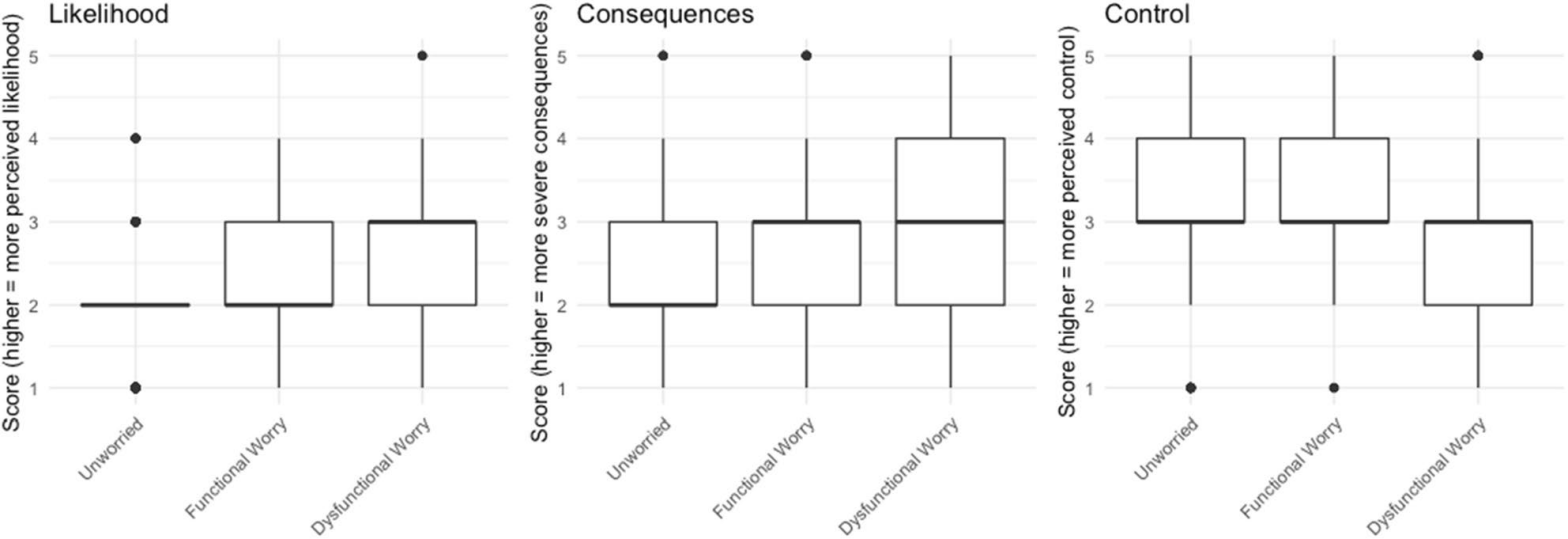
Perceived likelihood, control, and consequences between worry groups

Indeed, we find the unworried group have lower perceived likelihood and severity of consequences and higher perceived control, the functionally worried group are in the middle, and those in the dysfunctional worry group have the highest perceived likelihood and perceive the most severe consequences (Fig. 3).

To quantify, we set ‘dysfunctional worry’ as the reference category to see how these elements of risk perception are associated with membership of either functional or unworried groups in comparison (Table 8).

**Table 8:** Risk perception and worry group

	Dependent variable			
	Unworried	Functional Worry	Unworried	Functional Worry
	(1)	(2)	(3)	(4)
*Likelihood*	*0.455*	*0.781*	*0.538*	*0.883*
	(0.119)	(0.137)	(0.138)	(0.167)
	p = 0.000^***^	p = 0.071^*^	p = 0.00001^***^	*p = 0.457*
*Control*	*1.189*	*1.299*	*1.232*	*1.377*
	(0.098)	(0.117)	(0.115)	(0.145)
	p = 0.077^*^	p = 0.026^**^	p = 0.070^*^	p = 0.028^**^
*Severity*	*0.394*	*0.760*	*0.405*	*0.798*
	(0.096)	(0.105)	(0.117)	(0.133)
	p = 0.000^***^	p = 0.009^***^	p = 0.000^***^	p = 0.090^*^
Had COVID-19			0.816	1.578
			(0.249)	(0.374)
			p = 0.415	p = 0.223
Affected by COVID-19			0.868	0.751
			(0.122)	(0.155)
			p = 0.247	p = 0.064^*^
Age 25–44^a^			0.894	0.581
			(0.253)	(0.303)
			p = 0.660	p = 0.074^*^
Age 45–64^a^			1.030	0.565
			(0.338)	(0.406)
			p = 0.931	p = 0.160
Age 65+^a^			1.779	0.864
			(0.677)	(0.790)
			p = 0.395	p = 0.854
Female (ref: Male)			0.653	0.883
			(0.198)	(0.250)
			p = 0.032^**^	p = 0.618
White (ref: BAME)			0.995	0.938
			(0.276)	(0.336)
			p = 0.986	p = 0.849
Is keyworker			0.978	0.996
			(0.035)	(0.044)
			p = 0.529	p = 0.933
Cardiff^b^			1.344	1.310
			(0.517)	(0.647)
			p = 0.567	p = 0.677
Edinburgh^b^			1.246	0.916
			(0.477)	(0.615)
			p = 0.646	p = 0.887
Glasgow^b^			1.088	1.295
			(0.483)	(0.601)
			p = 0.861	p = 0.667
Leeds^b^			0.575	0.641
			(0.463)	(0.600)
			p = 0.232	p = 0.458
Liverpool^b^			1.199	1.274
			(0.487)	(0.606)
			p = 0.710	p = 0.690
London^b^			1.153	1.252
			(0.395)	(0.496)
			p = 0.719	p = 0.652
Manchester^b^			1.349	1.380
			(0.472)	(0.590)
			p = 0.527	p = 0.586
Newcastle^b^			0.836	0.996
			(0.552)	(0.706)
			p = 0.747	p = 0.996
			(0.536)	(0.634)
			p = 0.160	p = 0.078^*^
Constant	56.120	0.898	107.074	0.129
	(0.486)	(0.582)	(1.223)	(1.735)
	p = 0.000^***^	p = 0.854	p = 0.0002^***^	p = 0.239
Akaike Inf. Crit.	1767.581	1767.581	1346.572	1346.572

Multinomial logistic regression model estimated using R (package nnet). Source: Wave 3 of Policing the pandemic. Total *n* = 707

* p < 0.05, ** p < 0.01, *** p < 0.001

^a^Reference category: Age 16–24

^b^Reference category: Birmingham

Dysfunctionally worried group members perceive higher likelihood and more severe consequences of catching COVID-19 than both unworried and functionally worried group members, while also perceiving lower control over whether or not they will get exposed to the virus. Since these are perceptions, one approach that an intervention could take would be to consider messaging aimed at addressing these, maybe considering ways in which people might gain greater control over their risk of infection.

## Discussion

Our paper has approached worry about COVID-19 as something that can have both negative outcomes in terms of people’s mental health and emotional wellbeing, and positive consequences in terms of taking precautions and helping people protect themselves from the consequences of the virus. Analysing data from a panel study of attitudes and experiences with COVID-19 across ten UK cities, we classified people into groups of functional, dysfunctional, and no worry, based on previous development of such typologies in fear of crime literature. Using this method we found that dysfunctional worry (i.e. worry which affects quality of life, or leads to taking precautions which do not make the person feel safer or result in reduced quality of life) was associated with negative emotional outcomes which may affect people’s mental health. On the other hand, people who experienced functional worry, which is worry that encourages them to take precautions, did not have the same association with these negative outcomes. This suggests that, for some people, worry can be beneficial, and does not damage wellbeing. Similar to the relationship between previous victimisation and fear of crime, we found that having previous negative experiences as a result of COVID-19 such as loss of income or housing was associated with dysfunctional worry, but unlike crime victimisation, having had COVID-19 was associated with functional worry instead.

We also found, however, that none of the groups of worry were associated with either levels of compliance with lockdown measures, nor with subsequent re-engagement in these activities once the restrictions are lifted. It is instead likely that other factors are at play, such as the sense of a common fate, a shared identity, or acting for the common or the social good, centred around national sentiment towards the NHS for example.

Finally, one key difference between the worry groups was perceived risk, made up of the perceived likelihood of getting COVID-19, perceived severity of the consequences, and their perceived control over it. Future work could explore any causal relationship in order to inform targeted interventions that may help move people out of the dysfunctional worry group into the functional one and thus, it might be hoped, improving their quality of life and other outcomes. Interventions aimed at reducing worry should target those experiencing dysfunctional worry, possibly by exploring the effect that targeted messaging/communications campaigns might have on reducing perceived likelihood and severity of consequences, or increasing people’s perceived ability to control whether or not they contract the virus.

Of course, such targeted messaging should take into account all possible consequences, and specifically that the aim is to move people from dysfunctional to functional worry. We would want to eliminate the negative outcomes on wellbeing, but maintain the prosocial precautionary behaviour of functionally worried people. For example, it might be an intervention to trial whether identifying and messaging dysfunctionally worried people about ways in which they can gain some control over their risk of catching COVID-19, for example by talking about wearing masks (something they can control) in places where they do not perceive other people to be following social distancing guidelines (something they cannot control) to increase perceived control. A follow-up evaluation of such messaging might measure to what extent such messaging influences perceived control, and whether if it increases people’s sense of control this is associated with moving out of dysfunctional and into functional worry. We note that risk perception has to be understood in a systems context, as people belonging in one worry group or the other is likely influenced by a myriad of complex reasons (e.g. past experiences, existing circumstances, policies) and so on, but targeted (e.g. warning) messages can influence these perceptions within said contexts (Laughery and Wogalter 1997).

In light of the above findings, we suggest that future studies exploring worry about COVID-19 take into account this difference between functional and dysfunctional worry. Of course, we realise that asking many follow-up questions is not always possible due to resource and other constraints, so asking about worry and precautions might not always be feasible. In our data, we also explored a simplified classification, in which we do not take into account precautions, but instead divide people into functional or dysfunctional worry based on whether their worry reduces their quality of life or not. Using this simpler classification, we find the same relationships between dysfunctional worry and worse negative emotional outcomes, and the same differences in risk perception, alongside there still being no relationship between worry and compliance or re-engagement (see Appendix 2). While such a simplified definition loses some of the nuance of considering the effect of precautions (whether these made people feel safer and/or reduced their quality of life), in situations of constrained resources they should identify similar patterns to the more complete definition. Further work might pursue a more nuanced definition which considers the intensity and frequency of precautionary behaviours in the classification of dysfunctional and functional fear, distinguishing between active or passive behaviours in response to perceived threats that are proximal or distal within an individual’s environment, and whether a shift from active precautions to more passive precautions account for functional worry.

## Data Availability

Code for the analysis of this paper is available on GitHub here: https://github.com/maczokni/foc19analysis/. A project page for the Policing the Pandemic project is available on OSF here: https://osf.io/4vje3/. The pre-print of this paper is available on OSF here: https://osf.io/preprints/socarxiv/2kfs6..

## Appendix 1

Worry and precautionary activity.

- In the past 3 weeks, have you ever felt worried about getting COVID-19? (Yes/No).
- To what extent was your quality of life reduced by your worry about COVID-19? (Not at all—very much).
- Do you take any precautions against getting COVID-19? (Yes/No).
- To what extent do these precautions reduce your quality of life? (Not at all—very much).
- To what extent do these precautions make you feel safer? (Not at all—very much).

Catching COVID-19.

Research participants were asked whether they had had COVID-19 themselves or not, counting both confirmed and suspected cases as “Yes” they had had COVID-19.

Impact of COVID-19 on people’s lives.

We asked a series of questions designed to tap into how each person had been personally affected by COVID-19. These are:

- Lost your job/been unable to do paid work.
- Other member of your household lost their job or was unable to do paid work.
- Unable to pay bills.
- Evicted/lost accommodation.
- Unable to access sufficient food.
- Unable to access required medication.
- Somebody close to you is in hospital with Covid-19.
- You lost somebody close to you to Covid-19.

Here we simply sum all the cases where the person answered that ‘yes’ this had happened to them to attain a score of how much the person had been affected by COVID-19, where higher score means more severely affected.

Emotions.

To measure people’s emotions, we asked the following questions:

- Thinking about how you felt *yesterday*, overall, how strongly did you feel the following emotions? (Not at all—Very Strongly)
  - Anxiety.
  - Anger.
  - Loneliness.
  - Happiness.
  .
- Overall, how satisfied are you with your life nowadays? (Not at all satisfied—Completely satisfied).
- Overall, to what extent do you feel that the things you do in your life are worthwhile? (Not at all worthwhile—Completely worthwhile).

Compliance and reengagement.

In waves 1, 2 and 3 we measured what in the first two waves represented compliance with lockdown. We asked how often in the past 3 weeks research participants had:

- Socialised in person with friends or relatives whom you don’t live with.
- Went out for a walk, run, or cycle and spent more than a few minutes sitting somewhere to relax.
- Travelled for leisure (e.g. driven somewhere to go for a walk).

By the time of wave 3, these behaviours no longer broke lockdown because of its gradual ease. People in the UK were being encouraged to re-engage in some semblance of normal life–they were allowed to stop and rest during or after walking, running and cycling, they were allowed to travel for leisure, and they were allowed to socialise in groups of 6 (8 in Scotland). Thus, changes in self-reported behaviour between waves 2 and 3 represented the extent to which research participants were re-engaging. We calculated a re-engagement score by taking the score for the compliance item in wave 2 and subtracting it from the sum of the compliance items in wave 3. This means higher scores means more re-engagement: 0 is same engagement, negative is more engagement in wave 2 than wave 3 and positive is more participation in the activity in wave 3 than wave 2.

Knowledge of the virus.

- How would you rate your knowledge level on Covid-19? [1–5: bad, poor, fair, good, or excellent].

Perceived risk of sanction.

How likely is it that someone would get caught and sanctioned should they engage in each of the following behaviours during the Covid-19 outbreak? [1–5: Not at all likely, not very likely, neither likely nor unlikely, fairly likely, or very likely].

1. Socialised in person with friends or relatives whom you don’t live with.
2. Go out for a walk, run, or cycle and spend more than a few minutes sitting somewhere to relax.
3. Travelled for leisure (e.g. driven somewhere to go for a walk).

Expressive function of the law.

1. Do you think it was right or wrong to make social distancing a legal requirement?
  [1–5: completely wrong, wrong, undecided, right, or completely right].
2. By making it a legal requirement, the government sent the message that social distancing is important to fight the pandemic.
  [1–5: strongly disagree, somewhat agree, neither agree nor disagree, somewhat agree, or strongly agree].
3. Making social distancing a legal requirement helped to clarify what we should and should not be doing.
  [1–5: strongly disagree, somewhat agree, neither agree nor disagree, somewhat agree, or strongly agree].

Social norms.

Thinking about people socially distancing themselves to help prevent the spread of Covid-19, to what extent do you agree or disagree with the following statements?

1. Everybody should strictly follow social distancing to help prevent the spread of Covid-19.
2. Most people in my local community think it is the right thing to strictly follow social distancing to help prevent the spread of Covid-19.
3. Most people in my local community would disapprove if some individuals were not strictly following social distancing to help prevent the spread of Covid-19.
  [1–5: strongly disagree, somewhat agree, neither agree nor disagree, somewhat agree, or strongly agree].
4. How important is it to the National Health Service that everybody sticks to the guidelines on social distancing?
  [1–5: not at all important, not very important, moderately important, very important, or extremely important].

Duty to obey the police.

To what extent is it your moral duty to… [1–5: not at all my duty, somewhat not my duty, undecided, somewhat my duty, or completely my duty].

1. …obey the police.
2. …support the decisions of police officers, even if you disagree with them.
3. …do what the police tell you even if you don’t understand or agree with the reasons.

Normative alignment with the police.

To what extent do you agree or disagree with the following question about police in your local area? [1–5: strongly disagree, disagree, neither disagree nor agree, agree, or strongly agree].

1. I support the way the police usually act.
2. The police usually act in ways that are consistent with my own ideas about what is right and wrong.
3. The police stand up for values that are important for people like me.

Duty to obey the law.

Thinking about the law in the United Kingdom, to what extent do you agree or disagree with each of the following statements? [1–5: strongly disagree, disagree, neither disagree nor agree, agree, or strongly agree].

1. People should do what the law says.
2. A person who disobeys laws is a danger to others in the community.
3. Obeying the law ultimately benefits everyone in the community.

Normative alignment with the law.

And thinking about the law in the United Kingdom, to what extent do you agree or disagree with each of the following statements? [1–5: strongly disagree, disagree, neither disagree nor agree, agree, or strongly agree].

1. My own feelings about what is right and wrong usually agree with the laws that are enforced by the police and the courts.
2. The laws in my community are consistent with your own intuitions about what is right and just.
3. The laws of our criminal justice system are generally consistent with the views of the people in our community about what is right and wrong.

Risk perception.

To explore this, we consider people’s reply to three risk related questionnaire items adapted from fear of crime perceived likelihood questions (Jackson 2009):

- Perceived Likelihood: “How likely do you think it is that, in the next 3 weeks, you will catch COVID-19?”
- Perceived Severity of Consequences: “If you contracted COVID-19, how severe do you expect its consequences to be on your health?”
- Perceived Control: “To what extent do you feel able to control whether or not, in the next 3 weeks, you will catch COVID-19?”

## Appendix 2

### Results using the simplified classification

In this appendix we present the results above using the simpler classification scheme.

### Defining and measuring fear of COVID-19

In wave 2 just over one-third (34%) of people said that “No”, they had not worried about getting COVID-19 in the past 3 weeks. From those who said “Yes”, there was variation in self-reported frequency and intensity of worry. 35% of people who were worried experienced this “Once or twice” in the last 3 weeks, while 21% worried more than 10 times in this timeframe. On the last occasion, 58% said they “felt fairly worried” or “very worried”.

First, individuals were classified as unworried if they reported being unworried about catching COVID-19: it did not matter if they took precautions that made them feel safer, or if their quality of life was reduced by their precautions; if they reported being unworried they were simply classified as unworried.

To be classified in the functional worry group, respondents must have met three conditions: (a) they must have reported being worried about crime; (b) they must have taken precautions that made them feel safer; and (c) they must have judged their quality of life unaffected by either their worries or their precautions. Importantly, we assume that the worry process partly motivates these beneficial precautions; as Tallis and Eysenck 1994argue, worry can play a problem-solving role in people’s lives by stimulating action and helping them deal with uncertain future events. Finally, to be classified in the dysfunctional worry group, respondents must have reported being worried about COVID-19 but also that their quality of life was reduced by either their worries or their precautions (or both).

To generate the three groups, Tables 9 and 10 break down the sample. Overall 34% (n = 401, weighted n = 312) of respondents were unworried. The other two categories—functional worry and dysfunctional worry—are subsets of the remaining 65% (n = 699, weighted n = 596).

**Table 9:** Previous experience with COVID-19, and worry group (simple classification)

	*Dependent variable:*			
	Functional Worry	Dysfunctional Worry	Functional Worry	Dysfunctional Worry
	(1)	(2)	(3)	(4)
Had COVID-19	1.994 (0.226)	0.867 (0.189)	1.821 (0.276)	0.776 (0.228)
	p = 0.003^***^	p = 0.450	p = 0.030^**^	p = 0.268
Affected by COVID-19	1.035 (0.106)	1.611 (0.095)	0.931 (0.133)	1.617 (0.114)
	p = 0.742	p = 0.00000^***^	p = 0.589	p = 0.00003^***^
Age 25–44			1.094 (0.228)	1.175 (0.225)
			p = 0.693	p = 0.474
Age 45–64			2.158 (0.313)	1.673 (0.327)
			p = 0.015^**^	p = 0.116
Age 65+			2.236 (0.670)	2.327 (0.696)
			p = 0.230	p = 0.226
Female			1.287 (0.184)	1.295 (0.186)
			p = 0.171	p = 0.165
White			0.971 (0.255)	1.159 (0.256)
			p = 0.908	p = 0.564
Key worker			0.981 (0.034)	0.961 (0.033)
			p = 0.576	p = 0.229
Cardiff			1.150 (0.491)	1.122 (0.489)
			p = 0.776	p = 0.814
Edinburgh			1.857 (0.455)	1.377 (0.465)
			p = 0.174	p = 0.492
Glasgow			1.084 (0.452)	0.805 (0.461)
			p = 0.859	p = 0.637
Leeds			1.471 (0.459)	2.185 (0.435)
			p = 0.400	p = 0.073^*^
Liverpool			1.446 (0.459)	1.499 (0.449)
			p = 0.422	p = 0.368
London			1.295 (0.368)	1.274 (0.361)
			p = 0.483	p = 0.504
Manchester			1.281 (0.443)	0.979 (0.449)
			p = 0.577	p = 0.963
Newcastle			1.037 (0.527)	0.799 (0.536)
			p = 0.946	p = 0.676
Sheffield			1.846 (0.444)	0.591 (0.508)
			p = 0.168	p = 0.301
Constant	0.061 (0.881)	1.162 (0.728)	0.057 (1.182)	1.395 (0.991)
	p = 0.002^***^	p = 0.837	p = 0.016^**^	p = 0.737
Akaike Inf. Crit.	2302.165	2302.165	1721.537	1721.537

* p < 0.05, ** p < 0.01, *** p < 0.001

**Table 10:** Negative emotional wellbeing outcomes by worry group (simple classification)

	Dependent variable	
	Emotion score	
	(1)	(2)
Functional worry	0.968 (0.022)	0.985 (0.027)
	p = 0.145	p = 0.586
Dysfunctional worry	1.212 (0.022)	1.204 (0.027)
	p = 0.000^***^	p = 0.000^***^
Had COVID -19		0.982 (0.031)
		p = 0.550
Affected by COVID-19		1.056 (0.016)
		p = 0.0005^***^
Age 25–44		0.959 (0.029)
		p = 0.149
Age 45–64		0.889 (0.031)
		p = 0.0002^***^
Age 65+		0.816 (0.043)
		p = 0.00001^***^
Female		1.004 (0.022)
		p = 0.842
White		1.099 (0.039)
		p = 0.015^**^
Key worker		1.010 (0.004)
		p = 0.019^**^
Cardiff		0.956 (0.046)
		p = 0.329
Edinburgh		0.912 (0.048)
		p = 0.057^*^
Glasgow		0.977 (0.046)
		p = 0.621
Leeds		1.001 (0.046)
		p = 0.984
Liverpool		0.955 (0.048)
		p = 0.338
London		0.990 (0.048)
		p = 0.840
Manchester		1.011 (0.048)
		p = 0.826
Newcastle		1.026 (0.048)
		p = 0.590
Sheffield		0.910 (0.049)
		p = 0.056^*^
Constant	14.312 (0.016)	13.654 (0.134)
	p = 0.000^***^	p = 0.000^***^
Observations	1067	786
Log Likelihood	− 2479.504	− 1647.208
theta	177.845^*^ (105.666)	133,269.500 (1,263,562.000)
Akaike Inf. Crit.	4965.008	3334.417

* p < 0.05, ** p < 0.01, *** p < 0.001

Of these 65%, 4% (n = 34, weighted n = 25) took no precautions, 91% (n = 641, weighted n = 541) took precautions and felt safer as a result, and 1% (n = 5, weighted n = 6) took precautions but did not feel safer as a result (Table 9). We make this distinction because of the central role that beneficial precautionary activity plays in the functional/dysfunctional distinction.

Table 9 here.

Table 10 takes the categorisation process one step further by considering also whether the worry or the precautions had an effect on people’s quality of life. Overall 37% (n = 206, weighted n = 203) said their quality of life was not affected by either precautions or worry, 33% (n = 236, weighted n = 181) said they were affected by both, 12% (n = 82, weighted n = 64) said their quality of life was reduced by their worry but not the precautions, and 18% (n = 122, weighted n = 99) said this was the other way around (precautions but not the worry).

By cross-tabulating precautionary activity with levels of impact on quality of life, we can identify the functionally worried and the dysfunctionally worried. The cell to highlight is top-left (Table 10). This represents the functionally worried—the subset of the sample who were worried about COVID-19, who took precautions that made them feel safer, and whose quality of life was not reduced by either worry or precaution. The other three cells comprise the dysfunctional worry group.

Bringing this classification process to a close, we found that just over one-third (34% (n = 401, weighted n = 312)) of the sample were unworried, about one-in-five (36% (n = 355, weighted n = 331)) were functionally worried, and just less than a half (29% (n = 344, weighted n = 265)) were dysfunctionally worried (Table 11).

**Table 11:** Poisson model of self-reported non-compliance with social distancing

	Coeff.	Std error	P-value	95% CI	
Constant	0.26	0.81	0.42	− 0.36	0.87
Knowledge about COVID-19	− 0.10	− 1.59	0.11	− 0.23	0.02
Deterrence (perceived chance of police intervention in the event of non-compliance)^a^	− 0.01	− 0.25	0.80	− 0.11	0.08
Perceived legal legitimacy^a^	− 0.17	− 2.48	0.01	− 0.31	− 0.04
Perceived police legitimacy^a^	0.18	2.56	0.01	0.04	0.31
Social norms regarding social distancing^a^	− 0.47	− 6.71	0.00	− 0.61	− 0.34
Expressive function of the law^a^	− 0.22	− 4.87	0.00	− 0.31	− 0.13
Functional worry about catching COVID-19	− 0.29	− 2.77	0.01	− 0.50	− 0.09
Dysfunctional worry about catching COVID-19	− 0.16	− 1.45	0.15	− 0.37	0.06
Age gender interaction: 16–24 female^b^	− 0.41	− 2.41	0.02	− 0.75	− 0.08
25–44 male^b^	− 0.23	− 1.56	0.12	− 0.51	0.06
25–44 female^b^	− 0.47	− 3.46	0.00	− 0.74	− 0.20
45–64 male^b^	− 0.38	− 1.96	0.05	− 0.75	0.00
45–64 female^b^	− 0.98	− 4.57	0.00	− 1.40	− 0.56
65 + male^b^	− 0.91	− 1.29	0.20	− 2.29	0.47
65 + female^b^	− 0.97	− 1.46	0.14	− 2.28	0.33
City: Cardiff^c^	− 0.11	− 0.49	0.62	− 0.55	0.33
Edinburgh^c^	− 0.29	− 1.37	0.17	− 0.71	0.13
Glasgow^c^	− 0.12	− 0.56	0.57	− 0.52	0.29
Leeds^c^	0.10	0.48	0.64	− 0.30	0.49
Liverpool^c^	− 0.05	− 0.22	0.83	− 0.45	0.36
London^c^	0.23	1.46	0.14	− 0.08	0.53
Manchester^c^	− 0.04	− 0.21	0.84	− 0.43	0.35
Newcastle^c^	− 0.31	− 1.37	0.17	− 0.76	0.13
Sheffield^c^	− 0.25	− 1.08	0.28	− 0.70	0.20
None of these^c^	0.05	0.24	0.81	− 0.37	0.48

Poisson regression model estimated using Stata 15. Source: Wave 3 of Policing the pandemic. Total *n* = 1015

*OR* odds ratio, *CI* confidence intervals

* p < 0.05, ** p < 0.01, *** p < 0.001

^a^Component scores saved from principal components analysis

^b^Reference category: 16–24 male

^c^Reference category: Birmingham

### Previous experience

See Table 9.

### Emotions

See Table 10.

### Compliance and social distancing

See Tables 11 and 12.

**Table 12:** Linear model of change in self-reported ‘lockdown compliance’ between waves 2 and 3 [high scores = less ‘compliance’]

	Coeff.	Std error	P-value	95% CI	
Constant	1.27	2.11	0.04	0.09	2.46
Knowledge about COVID-19 at wave 2	− 0.01	− 0.10	0.92	− 0.30	0.27
Deterrence (perceived chance of police intervention in the event of non-compliance) at wave 2^a^	− 0.11	− 1.07	0.28	− 0.32	0.10
Perceived legal legitimacy at wave 2^a^	0.14	1.00	0.32	− 0.14	0.41
Perceived police legitimacy at wave 2^a^	− 0.02	− 0.16	0.87	− 0.31	0.27
Social norms regarding social distancing at wave 2^a^	− 0.12	− 1.07	0.28	− 0.34	0.10
Expressive function of the law at wave 2^a^	0.28	2.49	0.01	0.06	0.49
Functional worry about catching COVID-19 at wave 2	0.08	0.45	0.65	− 0.27	0.43
Dysfunctional worry about catching COVID-19 at wave 2	− 0.02	− 0.11	0.92	− 0.38	0.34
Knowledge about COVID-19 at wave 3	− 0.01	− 0.03	0.97	− 0.30	0.29
Deterrence (perceived chance of police intervention in the event of non-compliance) at wave 3^a^	− 0.17	− 1.73	0.08	− 0.36	0.02
Perceived legal legitimacy at wave 3^a^	− 0.19	− 1.26	0.21	− 0.48	0.10
Perceived police legitimacy at wave 3^a^	0.24	1.53	0.13	− 0.07	0.56
Social norms regarding social distancing at wave 3^a^	− 0.02	− 0.18	0.85	− 0.24	0.19
Expressive function of the law at wave 3^a^	− 0.29	− 2.64	0.01	− 0.50	− 0.07
Age gender interaction: 16–24 female^b^	0.40	1.16	0.25	− 0.28	1.09
25–44 male^b^	− 0.10	− 0.30	0.76	− 0.72	0.53
25–44 female^b^	0.27	0.91	0.37	− 0.31	0.85
45–64 male^b^	− 0.13	− 0.33	0.75	− 0.88	0.63
45–64 female^b^	− 0.50	− 1.41	0.16	− 1.19	0.20
65 + male^b^	− 0.58	− 0.64	0.52	− 2.37	1.20
65 + female^b^	− 0.09	− 0.13	0.90	− 1.47	1.29
City: Cardiff^c^	− 0.65	− 1.76	0.08	− 1.36	0.07
Edinburgh^c^	− 0.30	− 0.85	0.40	− 0.99	0.39
Glasgow^c^	0.01	0.03	0.98	− 0.69	0.71
Leeds^c^	0.63	1.74	0.08	− 0.08	1.33
Liverpool^c^	− 0.09	− 0.25	0.80	− 0.81	0.63
London^c^	0.14	0.48	0.63	− 0.43	0.72
Manchester^c^	0.47	1.31	0.19	− 0.23	1.16
Newcastle^c^	0.35	0.92	0.36	− 0.40	1.09
Sheffield^c^	0.46	1.23	0.22	− 0.27	1.19
None of these^c^	− 0.05	− 0.12	0.90	− 0.81	0.71

Linear regression model estimated using Stata 15. Source: Waves 2 and 3 of Policing the pandemic. Total *n* = 1980

*OR* odds ratio, *CI* confidence intervals

* p < 0.05, ** p < 0.01, *** p < 0.001

^a^Component scores saved from principal components analysis

^b^Reference category: 16–24 male

^c^Reference category: Birmingham

### Movement between groups

While most people have stayed in the same group between the two waves (68%), almost a third (32%) moved between categories (Fig. 2).

Unworried group have lower perceived likelihood and severity of consequences and higher perceived control, the Coping group are in the middle, and the Struggling group have the highest perceived likelihood and perceive most severe consequences.

## Footnotes

1 This was calculated by re-balancing the data where the tabulation of results becomes more than a simple counting process.

2 At the time testing was not widespread, and only of these 3 reported confirmed diagnoses.
